# Biosecurity and Vector Behaviour: Evaluating the Potential Threat Posed by Anglers and Canoeists as Pathways for the Spread of Invasive Non-Native Species and Pathogens

**DOI:** 10.1371/journal.pone.0092788

**Published:** 2014-04-09

**Authors:** Lucy G. Anderson, Piran C. L. White, Paul D. Stebbing, Grant D. Stentiford, Alison M. Dunn

**Affiliations:** 1 School of Biology, University of Leeds, Leeds, United Kingdom; 2 Environment Department, University of York, York, United Kingdom; 3 Centre for Environment, Fisheries and Aquaculture Science (Cefas), Weymouth, United Kingdom; Swansea University, United Kingdom

## Abstract

Invasive non-native species (INNS) endanger native biodiversity and are a major economic problem. The management of pathways to prevent their introduction and establishment is a key target in the Convention on Biological Diversity's Aichi biodiversity targets for 2020. Freshwater environments are particularly susceptible to invasions as they are exposed to multiple introduction pathways, including non-native fish stocking and the release of boat ballast water. Since many freshwater INNS and aquatic pathogens can survive for several days in damp environments, there is potential for transport between water catchments on the equipment used by recreational anglers and canoeists. To quantify this biosecurity risk, we conducted an online questionnaire with 960 anglers and 599 canoeists to investigate their locations of activity, equipment used, and how frequently equipment was cleaned and/or dried after use. Anglers were also asked about their use and disposal of live bait. Our results indicate that 64% of anglers and 78.5% of canoeists use their equipment/boat in more than one catchment within a fortnight, the survival time of many of the INNS and pathogens considered in this study and that 12% of anglers and 50% of canoeists do so without either cleaning or drying their kit between uses. Furthermore, 8% of anglers and 28% of canoeists had used their equipment overseas without cleaning or drying it after each use which could facilitate both the introduction and secondary spread of INNS in the UK. Our results provide a baseline against which to evaluate the effectiveness of future biosecurity awareness campaigns, and identify groups to target with biosecurity awareness information. Our results also indicate that the biosecurity practices of these groups must improve to reduce the likelihood of inadvertently spreading INNS and pathogens through these activities.

## Introduction

Invasive non-native species (INNS) are a primary driver of biodiversity loss and a major economic problem, with management and mitigation costing an estimated US$120 billion in the USA [1], US$6.3 billion in Australia [2] and US$2.6 billion in the UK each year [3]. Their ecological impacts range from habitat degradation, to competition with native species, to the introduction of pathogens and disease[4]–[6]. As the eradication of an established INNS is rarely possible [7], [8], preventative management is an important and cost effective control strategy [9]. To this end, the management and prevention of INNS introductions is recognised as a global priority for biodiversity conservation and is listed as one of the Convention on Biological Diversity's (CBD) Aichi biodiversity key targets for 2020 [10].

Freshwater ecosystems are particularly vulnerable to INNS [11]. They are exposed to a wide range of transmission pathways including fish stocking, the redirection of water supplies, release of boat ballast and bilge water, release of exotic and ornamental plant and animal species, and the transfer of recreational angling and boating gear between sites [11]–[16]. Recent research indicates that fishing, boating and leisure activities are collectively responsible for almost 40% of aquatic species introductions into Europe [17]. The management of vectors such as these is considered to be one of the most effective strategies to prevent introduction and spread of invaders since numerous INNS threats can be controlled simultaneously [18], [19].

In the UK, freshwater ecosystems contain seven of the UK Environment Agency's 10 ‘most wanted’ invasive species [20] and are thought to be threatened by a further 11 [17]. Despite the estimated 4 million recreational anglers [21] and 404,000 canoe owners [22] in the UK, these groups have received little attention with regard their potential role in the introduction and secondary spread of aquatic invasive species and pathogens.

Since many INNS can survive in damp environments for a number of days or even weeks (Table 1), the potential exists for their introduction and spread between catchments on wet equipment used by anglers and boaters [12], [23]. Prominent examples include the zebra mussel (*Dreissena polymorpha*) introduced to Ireland on the hulls of boats [26]; the pathogen *Aphanomyces astaci* (causative agent of crayfish plague) vectored in mud and on damp angling gear [23], [24]; and the killer shrimp (*Dikerogammarus villosus*) which is able to survive for up to 15 days on damp angling equipment [25].

**Table 1:** Approximate survival times of notifiable freshwater pathogens listed by the World Organisation for Animal Health (OIE) and freshwater INNS listed in the Environment Agency's 10 ‘most wanted’ invasive species or as one of the potential invaders threatening Great Britain and Ireland [17].

Species	Survival time outside host (pathogens) or in damp conditions (INNS)		Reference
PATHOGENS			
*Gyrodactylus salaris*	2–5 days	R	[53]
Koi herpes virus	3 days	P	[54]
White spot syndrome virus	3–4 days	R	[55]
*Aphanomyces astaci*	16 days	P	[56]
*Batrachochytrium dendrobatidis*	7 days	P	[57]
Amphibian ranaviruses	1 month	P	[58]
Infectious haematopoietic necrosis	1 month	R	[58]
Spring viraemia of carp	5 weeks	R	[59]
Viral haemorrhagic septicaemia	49 days	R	[60]
INVASIVE NON-NATIVE SPECIES			
Topmouth gudgeon (*Pseudorasbora parva*)	Minutes (fish)	P	N/A
Zebra mussel (*Dreissena polymorpha*)	3–5 days	P	[61]
Signal crayfish (*Pacifastacus leniusculus*)	3–7 days	P	[62]
Killer shrimp (*Dikerogammarus villosus*)	15 days	P	[25]
Floating pennywort (*Hydrocotyle ranunculoides*)	No data available	P	
Parrots Feather (*Myriophyllum aquaticum*)	No data available	P	
Chinese mitten crab (*Eriocheir sinensis*)	No data available	P	
Ponto-caspian shrimp (*Echinogammarus trichiatus*)	6 days	R	[63]
Quagga mussel (*Dreissena rortriformis bugensis*)	3–5 days	R	[61]
Mean survival time (where known)	15 days		

P  =  species or pathogen is already present, R  =  this species poses a significant threat to UK freshwaters.

The aim of this study is to investigate the biosecurity risk posed by recreational anglers and canoeists in the UK. Specifically, we aim to i) identify potential pathways of INNS introduction and spread, ii) identify ‘higher risk’ groups who should be targeted with biosecurity guidance and iii) provide a baseline against which to evaluate the effectiveness of future biosecurity advice.

## Materials and Methods

### Ethics Statement

The questionnaire satisfied the University of Leeds' guidelines on ethical conduct (Ethics reference BIOSCI 12-043). All data was collected, stored and analysed anonymously. Respondents were asked for two items of demographic data (age group and sex) but no data was collected that would enable any respondent to be identified.

An online questionnaire survey was conducted using Bristol Online Surveys software [26]. The secretaries of 316 angling clubs and 241 canoeing clubs in England were contacted from listings in the UK Environment Agency's 'Where to Fish?' guides, online angling club databases and the British Canoe Union's list of canoe clubs, and asked to circulate the questionnaire to their members.

Anglers and canoeists were asked about the type and frequency of angling/canoeing carried out. In order to gain a representative overview of how far each respondent typically travels to take part in their sport, they were asked to list the three sites that they fish/canoe at most recently and the three sites that they fish/canoe at most frequently. The six sites were geocoded into latitude and longitude coordinates with Python's *Geopy* toolbox, using the Google Maps API. The catchment that each site fell into was identified using the *Extraction* tool in the ArcGIS Spatial Analyst extension within the ArcGIS 10.1 [27] Geographic Information System software, according to the European Union Water Framework Directive catchment areas.

Respondents were asked about the equipment used during each trip, and how frequently it was i) dried and ii) cleaned after use; iii) which cleaning products were used, if any; and iv) whether equipment had been used overseas, and in which countries. Anglers were also asked about their use of live bait (invertebrates and fish). Canoeists were asked about the factors that influenced whether they cleaned and dried their equipment at the end of each trip. To do this, they were asked to score each of 6 factors on a likert scale from 1 (not important) to 5 (very important) depending on how these factors influenced their decision to clean equipment after use. The factors were: i) availability of a hose, ii) cost of cleaning products, iii) time taken to clean boat, iv) availability of information about how to clean boat and v) how dirty their boat appeared to be. Canoeists were also asked if they were aware of the ‘Check, Clean, Dry’ biosecurity campaign launched by the UK Department for Environment, Food and Rural Affairs (Defra) in 2010 to see whether there were differences in the biosecurity practices of those who had and had not heard of the campaign. Respondents were asked about their awareness of the biosecurity campaign on the last page of the online questionnaire having already answered questions about their actions in order to minimise potential bias.

The answer options for closed-format questions were determined through consultation with Environment Agency Fisheries Officers, UK Rivers Trusts and biosecurity experts from the Centre for Environment, Fisheries and Aquaculture Science (Cefas). A prototype version of the questionnaire was also piloted with 15 anglers and 12 canoeists to prevent any misunderstandings, to check that the online questionnaire worked effectively and to ensure that sufficient answer options had been provided in the closed questions. The full questionnaire is available as supplementary material (Questionnaire S1).

### Hazard Scores

In order to explore the relative biosecurity hazard posed by different groups of anglers and canoeists, respondents were scored against a set of criteria from 1 (low hazard) to 5 (high hazard) by interrogating them against a set of criteria (Table 2). The criteria were: the number of catchments visited (N), the frequency of the activity (F), the frequency of equipment cleaning (C) and the frequency of equipment drying (D). Individual hazard score  =  N*F*C*D.

**Table 2:** Scoring scheme for the criteria against which each individual was assessed in the hazard analysis.

Risk Factor	Description	Hazard Score
Frequency of angling	Once a month or less frequently	1
	Once every three weeks	2
	One a fortnight	3
	Once a week	4
	More than once a week	5
Number of catchments visited	1 catchment	0
	2 catchments	2
	3 catchments	3
	4 catchments	4
	5 or 6 catchments	5
Cleaning of equipment	After every trip	1
	Every 2–5 trips	2
	Every 6–10 trips	3
	Every 11+ trips	4
	Never	5
Drying of equipment	After every trip	1
	Every 2–5 trips	2
	Every 6–10 trips	3
	Every 11+ trips	4
	Never	5

Scores from 1–5 correspond to a hazard gradient from 1 (very low) to 5 (very high). As we were considering secondary spread between river catchments, respondents scored 0 if they said that they only visited one catchment.

By multiplying hazards together, interactions between factors were incorporated into the overall hazard score. As there is insufficient data available with which to inform the relative importance of the different risk criteria, we gave them equal weighting when calculating the hazard scores. As we were primarily investigating the potential role of anglers and canoeists in the secondary spread of INNS between UK catchments, anglers and canoeists who only visited one catchment scored zero. Regardless of how frequently they cleaned or dried their equipment, or how frequently they travelled, their total score would remain zero as they posed no likelihood of moving invasive species or aquatic pathogens to another catchment.

## Results

Fifty two angling clubs and 70 canoeing clubs circulated the online questionnaire to their members. In total, 599 canoeists and 960 anglers completed the questionnaire (response rates 17% and 25%, respectively).

Following best practice advice of White *et al.* [28], our sample was verified using demographic information to ensure that respondents were representative of the angling/canoeing communities. Of the 960 angler respondents, 98% were men with the highest proportion of respondents aged between 55 and 64 (31%) followed by 45 and 54 (22%) and 65+ (17%). To ascertain whether our sample was representative of the UK angling population, a Kolmogorov–Smirnov test was performed in ‘R’ [29] to compare the age distribution of angler respondents to the age distribution of holders of Environment Agency rod licences in 2011- a requirement of all UK freshwater anglers. No significant difference was detected between the ages of the two groups (K-S Test, D = 0.24, p>0.05), nor was there a significant difference between the sex ratios of the two groups, with 2% of angler respondents and 5% of rod license holders being female (K-S Test, D  =  0.3, p<0.05).

Seventy percent of the 599 canoeist respondents were men, a sex ratio which was not significantly different from British Canoe Union figures on the sex ratio of UK recreational boat users (British Canoe Union 2011) (D = 0.7, p>0.05). Respondents were from a broad range of age groups, with 35–44 year old and 45–54 year old groups with the greatest number of respondents (16.4% and 26%, respectively). Unfortunately, data on the age profiles of UK canoeists were unavailable.

Respondents were broadly spread across different angling and canoeing categories. Forty four percent of anglers were coarse anglers (typically pleasure anglers using rods from the bank and catching any freshwater fish other than game fish), 25% were game anglers targeting trout, 13% were match anglers (typically angling in heavily stocked commercial fisheries and frequently travelling to different sites to attend competitions), 7% were barbel anglers, 6% were game anglers targeting salmon and 5% were pike anglers. Almost half of canoeist respondents (47%) were recreational canoeists using rivers while 21% canoed on lakes, 19% were competitive canoeists, 11.9% were sea kayakers who also took part in freshwater canoeing and 1.5% were long distance touring canoeists.

### Potential for Secondary Spread

**Figure 1:**
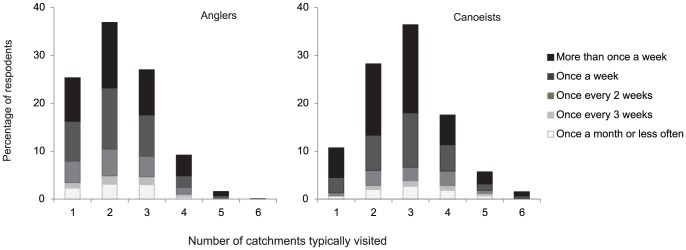
Typical number of UK catchments visited by canoeists and anglers. Shading shows the frequency with which respondents travelled between the catchments that they visited.

Anglers visited a mean of 2.25 different UK catchments (Figure 1). There was a significant difference in the number of catchments visited by categories of angler (ANOVA F_5,954_  =  9.56, p < 0.001). Posthoc (LSD) tests revealed that salmon anglers – who visited a mean of 2.79 catchments - travelled significantly further than any other group of angler (p<0.05). Canoeists visited a mean of 2.84 different catchments (Figure 1). There was a significant difference between the distances travelled by different categories of canoeist (ANOVA, F _4,594_  =  6.17, p < 0.001), however, posthoc (LSD) tests revealed that touring canoeists - who travelled to the highest number of catchments (mean 3.33) - did not visit significantly more catchments than other groups (p>0.05), while competitive canoeists did visit significantly fewer catchments than the other groups (mean of 2.53, posthoc tests p<0.05 when compared to each of the other groups).

**Figure 2:**
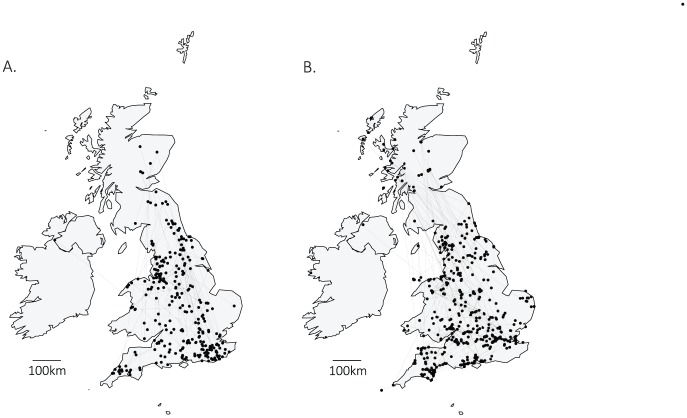
Maps showing the last three UK sites visited and by A) anglers and B) canoeists who visited more than one catchment within a fortnight and failed to clean or dry their kit between uses. Points show the sites and lines connect sites visited by an individual within a fortnight.

Sixty four percent of anglers and 79% of canoeists used their equipment in more than one catchment within a fortnight (Table 3), the mean duration that pathogens/INNS listed in Table 1 survived in damp conditions. Forty nine percent of canoeists and 12% of anglers visited more than one catchment within a fortnight and neither cleaned nor dried their kit between uses (Table 3). The geographic movements of these higher risk anglers and canoeists are displayed in Figure 2.

**Table 3:** Relative hazard scores for different categories of angler and canoeist.

Category	Median hazard score (0–625). Brackets indicate inter-quartile range.	% travelling to 2+ catchments	% travelling to 2+ catchments AND doing activity ≥ once per fortnight	% travelling to 2+ catchments AND doing activity ≥ once per fortnight AND not cleaning their equipment after every use	% travelling to 2+ catchments AND doing activity ≥ once per fortnight AND not drying their equipment after every use	% travelling to 2+ catchments AND doing activity ≥ once per fortnight AND neither cleaning nor drying their equipment after every use	% doing activity ≥ once per fortnight AND neither cleaning nor drying their equipment after every use AND using their equipment overseas
Game anglers (salmon)	40 (IQR 60)	88.5	80.3	63.9	19.7	19.7	8.2
Game anglers (trout)	30 (IQR 60)	82.6	68.6	56.8	14.0	13.6	8.1
Match anglers	20 (IQR 64)	74.2	72.6	52.4	15.3	12.9	8.9
Coarse anglers	16 (IQR 50)	69.6	56.8	45.1	11.9	11.2	9.0
Pike anglers	16 (IQR 52.5)	75.0	66.7	47.9	6.3	6.3	4.2
Barbel anglers	13.5 (IQR 60)	67.1	60.0	40.0	4.3	4.3	5.7
All anglers (n = 960)	20 (IQR = 50)	74.7	64.0	49.9	12.5	11.9	8.23
Competitive canoeists	100 (IQR 210)	85.6	83.8	55.0	79.3	52.3	36.0
River canoeists	96 (IQR 195)	89.4	74.6	54.1	68.6	50.9	31.1
Lake canoeists	80 (IQR 226)	87.2	77.6	50.4	68.6	48.8	19.2
Sea kayakers	75 (IQR 197.5)	97.2	85.9	47.9	67.6	40.8	18.3
Touring canoeists	60 (IQR 110)	100	88.9	44.4	77.8	44.4	11.1
All canoeists (n = 599)	80 (IQR = 220)	89.3	78.5	52.6	70.6	49.5	27.7

IQR  =  Interquartile range. Percentages show the co-occurrence of biosecurity hazards associated with potential transmission.

**Figure 3:**
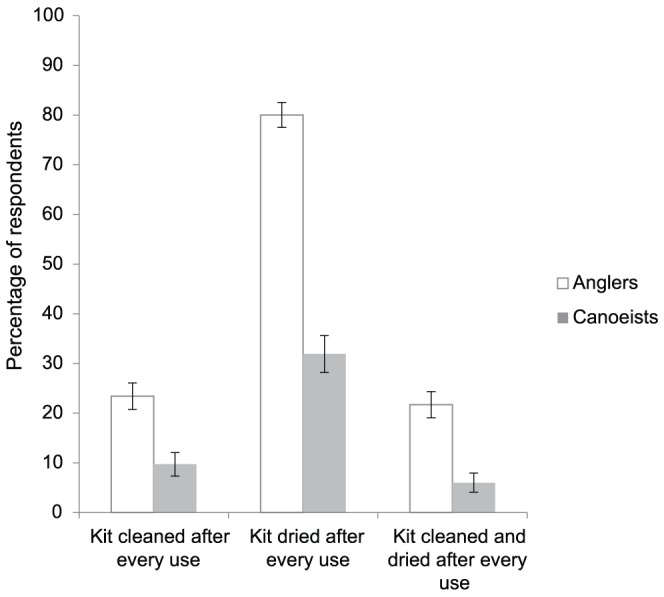
Percentage of anglers and canoeists who visited more than two catchments within a fortnight and who either cleaned, dried or cleaned and dried their equipment after every use. Error bars show 95% Confidence Interval.

Of the 614 anglers and 470 canoeists who used their equipment in more than one catchment within a fortnight, 22% of anglers and 10% of canoeists cleaned their kit after every use, 80% of anglers and 33% of canoeists dried their kit after every use and 21% of anglers and 6% of canoeists both cleaned and dried their kit after every use, the biosecurity advice recommended by Defra (Figure 3). Of the anglers who cleaned their kit after each use, 49% used tap water, 31% used disinfectant and 30% used detergent. Of the canoeists who cleaned their kit each time, 81% used tap water, 15% used detergent and 4% used disinfectant.

A large proportion of the anglers who travelled to more than one catchment within a fortnight without cleaning equipment after each trip used equipment associated with INNS/pathogen accumulation: rubber or felt soled waders (used by 36% and 4%, respectively) and keep nets (used by 25%).

### Hazard scores

Overall, anglers had lower hazard scores than canoeists, due to the higher proportion drying equipment, and the lower number of catchments visited (Table 3).

When different types of angler were compared, the median hazard scores of different groups were significantly different (Kruskal Wallis H  =  29.80; 5 df; p<0.001). Salmon anglers had the highest average hazard score, followed by trout anglers while pike and barbel anglers had the lowest (Table 4). In contrast, there was no significant difference between the hazard scores of different categories of canoeist (Kruskal Wallis H =  2.086; 4df; p>0.05). However, competitive canoeists had the highest mean hazard score and touring canoeists had the lowest (Table 3).

**Table 4:** The source and method of disposal of live bait by anglers.

		Bloodworms	Maggots	Earthworms	Fish
Source of bait	Bait dealer	22.1	4.8	7.1	2.4
	Catch own	8.8	10.3	29.4	90.5
	Purchased at fishery	4.4	0.8	0.0	2.4
	Tackle shop	64.7	84.1	63.5	4.8
Disposal method	Release into water	17.6	29.4	22.4	64.5
	Release onto land	7.4	7.9	21.2	0.8
	Freeze	27.9	18.3	18.8	27.4
	Throw in bin	7.4	4.0	5.9	3.2
	Take to next site	33.8	32.5	29.4	4.0
	Feed to garden birds	5.9	7.9	2.4	0.0

Figures show percentages for the source and disposal of each type of bait.

Canoeists were asked about the factors affecting whether they cleaned equipment after use. The availability of a hose was the most important factor (mean score 3.1 out of 5) followed by time availability (mean score 2.86 out of 5). The least important factors were the cost of cleaning products and the availability of information about how to clean equipment (both scored a mean of 1.9 out of 5). The 22 percent (130) of canoeist respondents who had heard of the ‘Check Clean Dry’ campaign exhibited biosecurity hazard scores that were 40% lower than those who had not (Kruskal-Wallis: H  =  10.99; df 1; p<0.001).

### Potential for Introduction to the UK

A large proportion of anglers (53%) and canoeists (46%) had used their equipment overseas, trips of between 260 km and 9500 km from their last site in the UK (excluding Ireland). The majority visited other countries within Europe (84% of anglers and 96% of canoeists) although 20% of respondents had used their equipment in North America and 7% in Australasia.

Within Europe, France and Ireland were the most popular angling destinations (visited by 17% and 16% of anglers, respectively). France and Austria were the most popular canoeing destinations (visited by 69% and 20% of travelling canoeists, respectively). Three percent of anglers had used their equipment in Norway where the salmon louse *Gyrodactylus salaris* poses a particular biosecurity threat to the UK [30].

Ninety nine percent of the 446 anglers who used their equipment overseas and went angling at least once a fortnight, failed to clean their equipment after every use, and 18% neither cleaned nor dried their equipment after every use. Moreover, 29% of the anglers who travelled overseas, fished at least once a fortnight and neither cleaned nor dried their equipment between uses used rubber waders, 5% used felt-soled waders and 19% used keep nets. Of the 241 canoeists who used their equipment overseas and at least once a fortnight and used their equipment overseas, 94% failed to clean it after every use, 71% failed to dry it after very use and 69% neither cleaned nor dried it after use.

### Use of Live Bait

Three hundred and seventy five of 960 angler respondents (39%) used live bait. Of those, 34% indicated using maggots, 34% indicated using bait fish, 23% indicated using earthworms and 18% indicated using bloodworms. The most commonly used bait fish were roach (*Rutilus rutilus*), rudd (*Scardinius erythrophthalmus*), perch (*Perca fluviatilis*), minnows (*Phoxinus phoxinus*) and gudgeon (*Gobio gobio*). The use of live bait was highest amongst pike anglers (47%) and barbel anglers (44%). Live bait use was lowest among trout anglers (36%).

**Figure 4:**
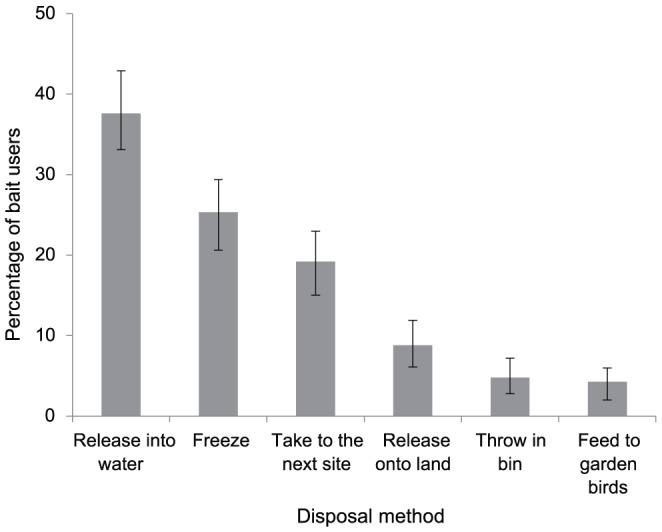
Disposal methods for live bait (fish and invertebrates) used by anglers. Error bars show 95% Confidence Interval.

Although the source varied between bait types (Table 4), the majority of bait users sourced bait from angling shops. Catching or collecting bait was the second most popular source of live bait. Of the 140 anglers who caught their own bait, fish and earthworms were most commonly sourced (77% and 17%, respectively) (Table 4). Baitfish was most commonly caught at the same site that the angler intended to use it (84%). However, 16% of anglers collected their bait at a different site from where it was to be used and 7% released unused bait which had been from a different site into the river/lake after use (Figure 4).

One hundred and forty bait users (37%) released unused bait into the water body at the end of their angling trip including bloodworms (9%) and baitfish (63%). Although the majority of anglers who released unused baitfish into the water had caught their fish at the same site, posing no biosecurity hazard, three had caught their baitfish at another site and one angler released bait fish sourced from a bait dealer. In addition, 9% of bait users released unused bait onto the land. This included 18 anglers who released unused earthworms on the river bank after use, all of which had been sourced from a different site to where they were released.

## Discussion

This is the first study exploring the potential biosecurity risk posed by anglers and canoeists in the UK. Our results highlight the fact that a high proportion of anglers and canoeists use their equipment frequently, and in multiple UK catchments, within the time that a range of INNS and aquatic pathogens can survive in damp conditions. This coupled with the low frequency with which anglers and canoeists clean and dry their equipment suggests that these groups may have the potential to act as vectors for their spread. The results are in accord with studies in North America which showed that anglers and boaters were travelling hundreds of kilometres between sites [31], [32] and were frequently transporting muddy waders [31], and the remnants of invasive aquatic plants on their boats [33], [34]. Within this research, we investigated the movement patterns of anglers and canoeists to evaluate the biosecurity risk that they would pose should they become contaminated with INNS or pathogens. We acknowledge that not all anglers and canoeists will use their equipment in a water body which has INNS present and of those who do, a low proportion of boats or equipment will become contaminated [35]. However, the large number of anglers (4 million) and canoe owners (404,000) in the UK and the frequency with which these groups appear to be using their equipment at different sites suggest that these groups pose an important pathway for the spread INNS should their equipment become contaminated.

### Potential for Secondary Spread

Fewer canoeists cleaned or dried equipment than anglers and only a small proportion of both groups used disinfectant. The low proportion of canoeists cleaning their equipment reflected the behaviour of boat users at Lake Simcoe, Canada where 19% rinsed their boats after use and only 3.2% allowed them to dry out for at least 5 days between uses [35]. Despite the seemingly high proportion of anglers drying equipment after use, complete drying is required in order to kill INNS and pathogens through desiccation [36], [37]. Considering the frequency with which anglers used their equipment it seems improbable that complete desiccation would have been achieved between trips.

The high frequency with which respondents took part in their activity may be an artefact of distributing the questionnaire to angling and canoeing clubs; themselves hubs of particular enthusiasts. Questionnaire surveys distributed in an ‘opt in’ manner can lend themselves to self-reporting bias [28], however due to data protection restrictions preventing us from contacting individuals in a more structured manner, this was an effective way of obtaining a large sample size. While care was taken to design an un-biased survey, we recognise that when people are asked about their individual actions in an environmental context, some ‘good behaviour’ bias may exist.

### Potential for Introduction to the UK

A high proportion of anglers and canoeists used their equipment overseas, primarily elsewhere in Europe. Moreover, a low proportion of both groups cleaned their equipment between uses – actions which may risk the inadvertent introduction of new aquatic invaders and pathogens from overseas. A number of the species mentioned in Table 1 such as the salmon ecto-parasite *Gyrodactylus salaris* and invasive amphipod *Dikerogammarus villosus* pose a hazard to native species in the UK, yet exist in regions of Europe where anglers and canoeists had used their own equipment and failed to clean it after each use (Table 3) [30], [38].

Countries such as New Zealand communicate a strong biosecurity message to water users at the international border, insisting that anglers and boaters check and disinfect their equipment before entering the country, as well as banning the use of hazardous equipment such as felt soled waders [39]. Considering the high proportion of UK water users who appear to use equipment overseas, there is likely to be a benefit from communicating a similar biosecurity message to these groups at UK ports and airports.

### Use of Live Bait

Release of bait fish from another site or from a bait dealer, the release of unused aquatic bloodworms from an unknown source and the disposal of earthworms on banksides after use pose clear biosecurity hazards. In the USA, the release of live bait has been the third largest source of non-native fish introductions [40] as well as being a major vector for the spread of invasive earthworms which have been associated with changing soil composition and local extinction of native plants [41].

The number of anglers who released bait fish having caught them at another site or from a bait dealer was much lower in our study than a comparable study in Maryland, USA, where 65% of anglers released unused bait which was frequently (and illegally) non-native crayfish [42]. Nonetheless, the release of unused bait fish by UK anglers is a recognised practice [43], and previous introductions of bait fish in the UK have been negatively correlated with the abundance of native fish species [43]. The release of bait is still therefore a potential route by which invasive species could be moved between UK catchments and one which needs controlling. Although more than a decade old, a study of anglers' attitudes to conservation in 2001 showed that only 19% of anglers saw the release of non-native bait fish as a conservation concern [44], despite the fact that failure to prevent the introduction of an invasive species risks prosecution under the Wildlife & Countryside Act, 1981.

The control of bait movement is also important to control the indirect spread of associated INNS including *A.astaci* zoospores, zebra mussels (*Dreissena polymorpha*) and Asian clams (*Corbicula fluminea*) which can be transported via the gastrointestinal tract of fish [45], [46] and may be moved between sites by anglers using live bait fish [47].

### Biosecurity

Our results indicate that in addition to the management of INNS vectors such as boat ballast water, and fish stocking, effective biosecurity practices are required to reduce the likelihood of INNS spread by recreational water users such as canoeists and anglers. In Europe, a large proportion (36%) of non-native species introductions are thought to have been due to fishing, boating and leisure activities [48]. Our results suggest that angler and canoeist activities pose a potential pathway for the spread of INNS or pathogens. As evidence indicates that pre-emptive management is effective at reducing the likelihood of aquatic INNS invasion via anthropogenic pathways [49], improving the biosecurity practices of these groups is important.

The lower proportion of canoeists cleaning/drying equipment after use as well as the higher proportion visiting three or more catchments was reflected in canoeists having higher biosecurity hazard scores than anglers. When canoeists were asked about factors affecting their cleaning/drying behaviours, the most important factor was the availability of a hose or cleaning station. The provision of more cleaning stations in ‘hot spot’ locations where boat and angling traffic is highest could therefore be fundamental to improving biosecurity practices.

Amongst anglers, we found game anglers, fishing salmonids, and match anglers to have the highest biosecurity hazard scores. Within the canoeing community, competitive canoeists and river canoeists had the highest hazard scores These groups were characterised by frequent canoeing/angling trips, often several times a week, visits to multiple catchments, and a low proportion of individuals cleaning and/or drying their equipment after every use. Biosecurity information should be targeted towards these groups of anglers, but we recognise that there was a lot of variation in biosecurity hazard scores within groups of canoeists so focusing biosecurity information towards specific types of canoeists may be less effective.

The UK Department for Food, Environment and Rural Affairs (Defra) launched a ‘Check, Clean, Dry’ biosecurity awareness campaign in 2010 in response to the first reports of the killer shrimp *Dikerogammarus villosus* in the UK [50]. The campaign was based on a biosecurity campaign in New Zealand designed to prevent the secondary spread of didymo (*Didymosphenia geminata*). In New Zealand, 80% of recreational water users were aware of the national ‘Clean, Check, Dry’ campaign and the spread of didymo appears to have slowed since the campaign was launched [39]. Our results indicate that the UK ‘Check, Clean, Dry’ biosecurity campaign has only reached a small proportion of water users to date. Nonetheless, canoeists in our study who reported awareness of the campaign also exhibited lower biosecurity hazard scores.

Public engagement is vital to effectively manage INNS [51]. It is therefore important to engage with recreational water users to raise awareness and regularly evaluate the effectiveness of biosecurity campaigns, not only to ensure that they are having the desired effect, but to provide evidence to the public that their actions make a difference [52]. Our results highlight the need to increase biosecurity awareness among recreational water users; however it is important to engage with these groups so that they continue to enjoy their sport whilst taking biosecurity into account. We have provided an important baseline against which to monitor the effectiveness of future biosecurity awareness campaigns. Our data also identify groups who pose a higher biosecurity hazard, and should therefore be targeted as a priority.

Finally, our data on equipment use can inform experiments that evaluate the effectiveness of different decontamination measures to prevent the survival of INNS and pathogens on angling and canoeing equipment and our spatial data can used to parameterise network models to identify hotspot locations to target with biosecurity control measures.

## Acknowledgments

The authors would like to thank fisheries officers from the Environment Agency for helpful comments about UK angling practices; epidemiologists from the Centre for Environment, Fisheries and Aquaculture Science (Cefas) for expertise on aquatic biosecurity hazards; and an anonymous reviewer for helpful comments on the manuscript.

## Supporting Information

Questionnaire S1 **This table outlines the questions which appeared in the online questionnaire distributed to canoeists and anglers using Bristol Online Surveys software.**
(DOCX)
