# Supplementary material for: Biosecurity and Vector Behaviour: Evaluating the Potential Threat Posed by Anglers and Canoeists as Pathways for the Spread of Invasive Non-Native Species and Pathogens
Source: PLoS One. 2014 Apr 9;9(4):e92788. doi: 10.1371/journal.pone.0092788 (PMC3981671; doi:10.1371/journal.pone.0092788)
Supplement: Questionnaire S1 — This table outlines the questions which appeared in the online questionnaire distributed to canoeists and anglers using Bristol Online Surveys software. (DOCX) [file pone.0092788.s001.docx]

**Questionnaire S1**

This table shows the structure of the questionnaire which was hosted online using Bristol Online Surveys software. Each section of the questionnaire (separated here with grey rows) was on a new webpage and respondents could not skip back to change previous answers having completed a section. The questionnaire was separated into two versions: one aimed at canoeists/kayakers and a second version aimed at anglers. The “angler only” questions only appeared in the anglers’ version and the “canoeists/kayakers only” questions only in the canoeists’ version, as highlighted below.

| **Demographic information** |  |
| --- | --- |
| Age | - under 18 - 18-24 - 25-34 - 35-44 - 45-54 - 55-64 - 65+ |
| Sex | Male/Female |
| Which type of canoeing/angling do you do most frequently? | Selection from a list |
| **Movement patterns** | |
| How frequently do you go angling/canoeing? | - More than once a week - Once a week - Once every 2 weeks - Once every 3 weeks - Once every month - Once every 2 months - Once every 3 months - More than once every 3 months |
| Please enter the first three or four digits of your postcode (This will enable us to estimate how far different water users travel to take part in their activities. Your location will remain anonymous) | Open answer |
| Please list the 3 sites you went angling at **most recently**. | Site name______ Nearest town______ County_______  Site name______ Nearest town______ County_______  Site name______ Nearest town______ County_______ |
| Please list the 3 sites that you go angling/canoeing at **most frequently** | Site name______ Nearest town______ County_______  Site name______ Nearest town______ County_______  Site name______ Nearest town______ County_______ |
| Have you ever used your own angling equipment/canoe outside the UK? | Yes/No |
| If yes, which countries? | Open answer |
| **Equipment use** | |
| Which of the following items of equipment do you use? | Multiple selections from list – specific to angling/canoeing |
| Where do you store your equipment between trips? | - Indoors - Outhouse or garage - Outdoors |
| If you use waders or a keep net, how long do you typically keep them in the water for? (anglers only) | ___hours (waders)  ___hours (keep net) |
| Do you ever clean you equipment between trips? | Yes/No |
| If yes, how frequently? | - After every trip - After 2-5 trips - After 6-10 trips - After 11+trips |
| If yes, what do you use? | - Water - Detergent - Disinfectant - Other (please state) |
| Do you ever dry your equipment completely between trips? | Yes/No |
| If yes, how frequently? | - After every trip - After 2-5 trips - After 6-10 trips - After 11+trips |
| **Anglers only** |  |
| Do you ever use live bait? | Yes/No |
| If yes, what type of bait do you use? | Multiple selections from list |
| If yes, where do you source your bait from? | Multiple selections from list |
| If yes, what do you do with your bait at the end of your angling trip? | Multiple selections from list |
| **Canoeists/kayakers only** |  |
| How important are the following factors when deciding whether to clean your kayak/canoe and equipment after a trip. | *Please rate the following from 1 to 5 (1 = not at all important, 5 = extremely important)*   - The availability of a hose/cleaning station - The cost of cleaning equipment - The time it takes to clean equipment - The availability of information about what to do - How clean your kayak/canoe looks at the end of your trip |
| **Check, Clean, Dry** |  |
| Have you heard of the “Clean, Check, Dry” campaign in the UK? | Yes/No |
